# De novo metatranscriptomic exploration of gene function in the millipede holobiont

**DOI:** 10.1038/s41598-022-19565-y

**Published:** 2022-09-28

**Authors:** Puspendu Sardar, Vladimír Šustr, Alica Chroňáková, František Lorenc, Lucie Faktorová

**Affiliations:** 1 Institute of Soil Biology, Biology Centre AS CR, Na Sádkách 7, 370 05 České Budějovice, Czech Republic; 2 Cambridge Institute of Therapeutic Immunology and Infectious Disease (CITIID), Department of Medicine, Cambridge Biomedical Campus, University of Cambridge, Cambridge, CB2 0AW UK; 3 Department of Food Biotechnologies and Agricultural Products’ Quality, Faculty of Agriculture and Technology, University of South Bohemia in České Budějovice, Studentská 1668, 370 05 České Budějovice, Czech Republic

**Keywords:** Classification and taxonomy, Functional clustering, Gene ontology, Protein function predictions, Sequence annotation, Microbial communities, Computational biology and bioinformatics

## Abstract

Invertebrate–microbial associations are widespread in the biosphere and are often related to the function of novel genes, fitness advantages, and even speciation events. Despite ~ 13,000 species of millipedes identified across the world, millipedes and their gut microbiota are markedly understudied compared to other arthropods. Exploring the contribution of individual host-associated microbes is often challenging as many are uncultivable. In this study, we conducted metatranscriptomic profiling of different body segments of a millipede at the holobiont level. This is the first reported transcriptome assembly of a tropical millipede *Telodeinopus aoutii* (Demange, 1971), as well as the first study on any Myriapoda holobiont. High-throughput RNA sequencing revealed that *Telodeinopus aoutii* contained > 90% of the core Arthropoda genes. Proteobacteria, Bacteroidetes, Firmicutes, and Euryarchaeota represented dominant and functionally active phyla in the millipede gut, among which 97% of Bacteroidetes and 98% of Firmicutes were present exclusively in the hindgut. A total of 37,831 predicted protein-coding genes of millipede holobiont belonged to six enzyme classes. Around 35% of these proteins were produced by microbiota in the hindgut and 21% by the host in the midgut. Our results indicated that although major metabolic pathways operate at the holobiont level, the involvement of some host and microbial genes are mutually exclusive and microbes predominantly contribute to essential amino acid biosynthesis, short-chain fatty acid metabolism, and fermentation.

## Introduction

Millipedes, along with other soil invertebrate decomposers, affect nutrient recycling in terrestrial ecosystems. Millipedes ingest around 5–25% of the annual leaf litter in some habitats^1^. The digestion process in millipedes is rather complex considering both autochthonous and microbial digestive activities^2^. Intestinal organisms in the millipede gut are rich and diverse, including archaea, bacteria, rhizobia, actinobacteria, filamentous fungi and yeasts, nematodes, and ciliates^1^. The millipede digestive tract is a straight tube^3^ without hindgut paunches. Experimental data supports the existence of a symbiotic interaction between the host digestive system and intestinal microbiome for lignocellulose degradation in millipedes^4^. However, the functional importance of gut microorganisms for millipedes has not been fully investigated^5^. In some arthropods microbial symbionts provide additional functions to the host: they detoxify ingested compounds, synthesize essential nutrients, fix atmospheric nitrogen, recycle nitrogenous wastes, promote host tolerance to extreme conditions, and provide protection from pathogenic microbes^6^. However, the contribution of gut microbiota to the digestion and organic matter transformation during passage through the millipede gut is still not sufficiently elucidated. Lynn Margulis and René Fester introduced the term “holobiont” in 1991^7^ to describe a simple biological entity comprising a host and a single inherited symbiont, and later extended it to the host and its associated microbiota. Genomes of a host and its microbiota are collectively referred to as hologenome, often overrepresented by microbial genes; for example there are about 20,000 genes in the human genome, but its hologenome contains > 33 million genes from its microbiota^8,9^. Bredon and colleagues referred to the holobiont concept in the context of the mutualistic interaction between the host and associated intestinal microbiota in lignocellulose degradation in terrestrial isopods^10^.

Besides the microbial part of the millipede hologenome, the millipede genome itself represents an interesting object of attention because of its unique gene regulation machinery. The millipede genome shares ancestral features with deuterostome genomes including humans, and genomic machinery, such as *Hox3*, *Xlox*, Argonaute proteins, and microRNAs that have undergone changes and led to unique adaptation during myriapod evolution^11^. As a means of chemical defense, millipedes produce diverse chemical cocktails of secondary metabolites^11,12^. These unique properties make millipedes an ideal model system to study body plan evolution, gene regulation, adaptation and chemical biosynthesis. Cultivation and imaging techniques provide useful information about the intestinal microbiota of invertebrates^1^, however, these methods are limited because many host-associated microbes are uncultivable outside their hosts. Molecular studies based on amplified ribosomal DNA-restriction analysis, fingerprinting, denaturing gradient gel electrophoresis, and cloning provided additional, but still limited, insights into the microbiota associated with some millipede species^13,14^. Nowadays omics techniques complemented by RNA sequencing and metatranscriptomics are utilized in studies of invertebrate–microbial interactions^15,16^, providing a large amount of information at the holobiont level^10,17,18^. Metatranscriptomics has been successfully applied to unravel the synergistic functions of genes of both host and microbial origin^19^, and used to describe digestive processes of detritivorous and xylophagous arthropods often hosting lignocellulolytic microbiota; mainly in beetles^6,20,21^ and terrestrial isopods^10^. Despite their taxonomic diversity, ecological importance, and unique genomic features, only three assembled millipede genomes are currently available: two chromosomal-level genomes of the millipedes *Helicorthomorpha holstii* (Pocock, 1895)^11^ and *Trigoniulus corallinus* (Eydoux and Souleyet, 1842)^11^ and a draft genome of the millipede *Trigoniulus corallinus*^22^. The few studies that have addressed transcriptome level analysis of millipedes have mostly dealt with phylogenomics^12,23,24^ and genome architecture^11^. To date, no comparative study on the host-microbiome interactions in a millipede holobiont has been published. Only a pre-print manuscript^25^ using shotgun nanopore sequencing described Proteobacteria, Bacteroidetes, and Firmicutes as major taxa in the gut of the spirobolidan millipede species *Anadenobolus monilicornis* (Porat, 1876).

In this study, we used the tropical millipede *Telodeinopus aoutii* (Demange, 1971) as our focal species. *T. aoutii* belongs to a group of large soil decomposers (Spirostreptida) that consume leaf litter, dead wood, fruits, and vegetables, and are naturally distributed in dry tree savannas with distinct rainy and dry seasons. The histology and ultrastructure of the digestive tract^26,27^ and the chemical composition of defensive secretions^28^ have been studied in this species to date. The large body size of some tropical species of millipedes facilitates sampling from different gut sections and compartments^2^. The current study was designed at the holobiont level, targeting the gene expression of both the millipede host and its intestinal microbiota. At the same time, the study aimed to assign the putative functions of expressed genes in different gut sections (foregut, midgut, and hindgut) and non-gut tissues. This study further attempted to verify the completeness of host transcriptome assembly, to assess the taxonomic and functional distribution of genes in the holobiont. Several general hypotheses were tested: (i) there is a difference in the active functional genes in the holobiont between host and symbionts, (ii) the ratio of symbiont- or host- derived active genes differs in different body parts, and (iii) symbionts are essential for host nutritional processes or the metabolism and ecological services of the whole holobiont organism.

## Materials and methods

### Experimental animals

Specimens of *Telodeinopus aoutii* (Diplopoda, Spirostreptida, Spirostreptidae) were obtained from pet shops in the Czech Republic and kept in laboratory conditions for several months. Millipedes were reared in plastic boxes (60 × 30 × 20 cm) at 25 °C on forest floor substrate with peat, rotten wood, and a mixture of leaf litter (maple, oak, and beech). The box lids were ventilated, and the floor substrate was regularly moistened with tap water. Leaf litter was the main food source of the millipede. Pieces of cucumber, pears, banana, apple, and cabbage were used as additional food sources. Cuttlebone powder and aquarium fish food were added to the floor substrate as the calcium and nitrogen sources. Individuals taken directly from the boxes and with full digestive tracts were used for gut dissection and subsequent analyses.

### Sample collection, RNA extraction, and transcriptomic sequencing

Four individual organisms were cleaned by RNase AWAY (Sigma-Aldrich) and euthanized by deep freezing at − 80 °C for 10 min. Shortly after during partial thawing, millipedes were dissected. The intestine was carefully removed and divided into foregut, midgut, and hindgut, under sterile conditions. For all individuals, total RNA was extracted with RNeasy Power Soil Total RNA Kit, QIAGEN from three distinct gut compartments (i.e., foregut: FG, midgut: MG, and hindgut: HG), and the rest of the body remained after dissection (non-gut: NG). Thus, four body segments of four biological replicates produced a total of 16 metatranscriptomic libraries. The crude RNA was precipitated by glycogen-ethanol-acetate solution and resuspended in DEPC water (Top-Bio, Prague, Czech Republic). DNA was digested using a TURBO DNA-free Kit (ThermoFisher Scientific) and purified using an AllPrep DNA/RNA Micro Kit (QIAGEN). DNA digestion efficiency was verified by amplifying 16S rRNA genes, and RNA quantity and quality were evaluated by RNA gel electrophoresis on an RNA Nano Chip (Agilent 2100 Bioanalyzer) (Supplementary Table S1). All RNA samples were stored at − 80 °C until shipment to the sequencing laboratory at the UIC’s Sequencing Core, Chicago, IL, USA. RNA-Seq libraries were constructed from approximately 120 ng of total RNA. For removing rRNA, a method described previously^29^ was followed with modifications that enabled depletion of both the host and microbial rRNA simultaneously. According to the manufacturer’s instructions, libraries were prepared using the Zymo-Seq RiboFree Total RNA Library Kit from ZYMO RESEARCH. RNA-Seq libraries were sequenced on an Illumina platform (2 × 100 bp paired-end Illumina MiSeq). The corresponding sequence files were deposited in NCBI’s Sequence Read Archive (BioProject PRJNA749320).

### Sequence processing and de novo transcriptome assembly

All sequences were processed using open sources, free software, and custom scripts (BASH, Perl, Python, and R) locally under Linux Ubuntu operated computers. An initial quality assessment of the sequencing reads was performed using the FastQC tool (http://www.bioinformatics.babraham.ac.uk/projects/fastqc/). Adapters were searched and removed from the Illumina reads using Trimmomatic (v0.38)^30^. Reads were re-imported to FastQC after the trimming process to check for the overall quality. The program SortMeRNA (v2.1)^31^ was used to remove rRNA reads from sequencing datasets using the following databases: SILVA 16S and 23S for archaeal and bacterial rRNA, SILVA 18S and 28S for eukaryotic rRNA, and Rfam for 5S and 5.8S. Due to the unavailability of the reference genomes and transcriptomes of *T. aoutii* in public databases, transcripts were assembled by de novo. De novo transcriptome assembly was performed with Trinity (v2.10.0)^32^. The following parameters were used for the de novo assembly: *--seqType fq –sample_files* < *description_of_input_sample_fastq_files* > *--CPU 50 --max_memory 400 G --min_contig_length 150*”. A default k-mer length of 25 was used for the assembly preparation. During the assembly process an internal quality trimming and in silico normalization of reads were done with default parameters within Trinity using Trimmomatic^30^ and the Trinity script *insilico_read_normalization.pl*, respectively. Initial assembly statistics were computed using *TrinityStats.pl* within the Trinity package.

### Assembly optimization, filtering, and completeness

The proportion of reads mapped to the assembly was assessed by mapping the reads back to their corresponding assemblies using Bowtie2 (v2.3.4.1)^33^. The probability of obtaining spurious and redundant transcripts was reduced by generating a set of non-redundant representative transcripts using the CD-Hit package^34^ with an identity threshold of 95%. Parameters set for clustering non-redundant transcripts were as follows: *cd-hit-est -c 0.95 -n 10 -M 60000 -T 18*. Transcriptome completeness was assessed using the Benchmarking Universal Single-Copy Orthologs (BUSCO) (v4.0.6)^35^, referring to core arthropod genes (arthropoda_odb10).

### Transcript quantification, normalization, and differential expression analysis

To quantify transcript abundance, we used a mapping-based method using the Salmon tool^36^ in the Trinity package with *align_and_estimate_abundance.pl* script by mapping the reads from each biological replicate against the assembly from the corresponding body segment (i.e. either FG, MG, HG, or NG). The above procedure yielded normalized expression values for the expression of a particular transcript in the sample, measured as Transcripts Per Million (TPM)^37^. TPM indicates the chance of finding a gene when randomly sampling one million genes in the metatranscriptome, thus accounting for both gene length and sequencing depth. After estimating the transcript abundance for each biological replicate in TPM, we merged the quasi-map indices and constructed two matrices using *abundance_estimates_to_matrix.pl* script, one containing the estimated transcript counts and the other containing the cross-sample normalized TPM expression values using the trimmed mean of M values (TMM) method^38^. The TMM gene expression matrix was used to obtain the expression level of each transcript by ExN50 analysis. The precomputed transcript count matrix was used for differential expression (DE) analysis using *run_DE_analysis.pl* script which uses Bioconductor package DESeq2^39^ for statistical analysis and identification of significantly differentially expressed transcripts in the data set. The results from DE analysis of pairwise comparison among different millipede body segments were checked. Only the *p*_adj_ < 0.05 (after false detection rate correction) values were used for further analysis. Venn Diagrams^40^ with the differentially abundant transcripts were plotted using the *library *(“*VennDiagram*”) in R^41^.

### Taxonomic assignment, community profiling, and functional annotation

Open reading frames (OFRs) with a minimum length of 200 amino acids were predicted with TransDecoder (v5.5.0)^42^. For the taxonomic assignment of the predicted ORFs, searches against the NCBI Non-Redundant protein database (1 July 2020) were performed using BLASTp^43^ in DIAMOND^44^ with the “--more-sensitive” mode with an E-value cut-off of 1e^−5^. The BLAST outputs were then imported into MEGAN6 software (v6.19.5)^45^ for the taxonomic assignment using the NCBI taxonomy database^46^ (July 2020). Each ORF was thus assigned to the most accurate taxonomic rank (i.e., kingdom, phylum, class, order, family, genus, and species) based on the lowest common ancestor (LCA) algorithm. To ensure that all the sequences were assigned to the most accurate taxonomic rank, two control conditions were implemented into the above-mentioned taxonomic assignment steps: (i) ORFs from contigs were searched against the Non-Redundant Protein database restricted to “Archaea”, “Bacteria”, “Ciliophora”, “Fungi”, “Nematoda”, and “Arthropoda” individually using BLASTp; (ii) BLAST outputs were then imported into MEGAN6 based on the LCA algorithm using the NCBI taxonomy database and all contigs associated with an ORF assigned to Arthropoda were filtered as millipede genes (host) while contigs associated with bacteria, archaea, and fungi were considered as the microbial genes. Although we computed the contigs associated with Ciliophora and Nematoda, we did not consider them for further analysis. Contigs assigned to the virus were filtered out and not considered for the functional annotation. Due to the simple anatomy of millipedes, the gut-associated microbes were considered the major active microbial pool of the millipede host. Therefore, any non-gut related microbial reads were disregarded in this study. The command-line version of eggNOG mapper v2^47^ was used for the functional annotation using precomputed orthologous groups and phylogenies from the eggNOG database^48^ with “one-to-one” orthology assignment. The use of orthology instead of traditional homology searches (i.e., BLAST searches) for the functional annotation allows for higher accuracy. It eliminates the chance of transferring annotations from close paralogs^47^. Data were then analyzed according to gene ontologies (GO IDs terms defined by the Gene Ontology Consortium)^49,50^ using: GOSlimViewer^51^, AmiGO^52^, and KEGG^53–55^. The following databases were used for the comparative analysis of gene functioning among the gut segments and the taxonomic origin of the genes (i.e. host and microbiota): SEED^56^, ENZYME database^57^, InterPro^58^, and eggNOG. Normalized gene counts were calculated using the Bray–Curtis dissimilarity matrix^59^ and used for the principal coordinate analysis (PCoA). Data from metabolic pathways were visualized using KEGG Mapper^60^. The ggplot2 (https://github.com/tidyverse/ggplot2) and Circos^61^ packages were used in R for plotting the data and presentation purposes. The hypothesis of equal distribution of the observed parameters among different body parts, host and holobiont microbiota was tested using nonparametric tests (Χ^2^ test of expected and observed frequencies) in Statistica v6.0.

## Results and discussion

### De novo transcriptome assembly

**Figure 1:**
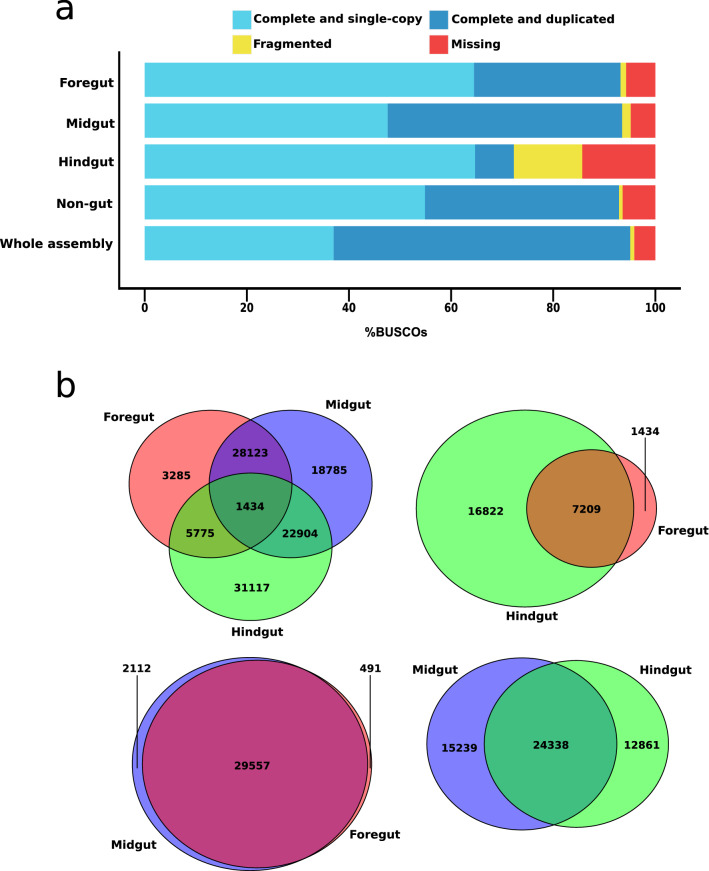
The assembly completeness and differential expression (DE) of transcripts are assessed in different body segments and whole assembly (combining all body segments). (**a**) Cumulative percentage of orthologs inferred from the BUSCO search using core Arthropoda genome. Complete orthologs can be either single-copy or duplicated. Incomplete orthologs are considered fragmented and, in the case of non-matching orthologs from the database, denoted as missing. (**b**) Venn diagram representing the DE analysis of transcripts from foregut, midgut, and hindgut. The Venn diagram numbers indicate either shared or unique expression data of different gut segments.

Four biological replicates from three sections of the digestive tract and non-digestive tissues of the millipede *T. aoutii* were processed to produce a total of 16 libraries to build the holo-transcriptome. Samples from different body parts enabled us to compare transcripts of local populations of symbiotic organisms in a single host species. These samples represented a total of 0.55 billion paired-end (PE) reads (Supplementary Table S2) in four assemblies representing the four body segments of *T. aoutii*. The assemblies resulted in 122,095 to 488,650 contigs with contig N_50_ ranging from 617 to 2337 bp in different body parts (Table 1) and displayed good completeness since more than 90% of the complete genes from the arthropod core genome were represented by FG, MG, and NG, and 72.3% of these genes were present in HG (Fig. 1a). Of the 1013 single-copy orthologous arthropod genes in the OrthoDB^62^, 95.1% of the complete genes (375 single-copy genes and 589 duplicated) were represented by the transcriptome comprising all body segments combined in one assembly of *T. aoutii*. This high duplication is probably the result of assembling transcriptomes from different body segments of the same species together. The transcriptome assembled from different body segments separately represented a relatively higher number of single-copy genes than the assembly comprising all the segments together. Among all the segments, assembly from the MG represented the highest completeness at 93.5% (482 single-copy and 465 duplicated genes), followed by FG at 93.2% (653 single-copy and 291 duplicated genes), NG at 92.9% (556 single-copy and 385 duplicated genes), and HG at 72.3% (655 single-copy and 77 duplicated genes). Although HG represented the lowest completeness among all the segments, it also contained the highest number of single-copy genes.

**Table 1:** De novo transcriptome assembly statistics, contig N_50_ number, non-redundant contig number, and predicted ORFs in the different gut and non-gut tissues in the *T. aoutii*. *N/A* not analyzed.

	FG	MG	HG	NG
Total assembled bases	113,974,610	331,200,029	82,252,590	198,222,627
Total contigs	122,095	488,650	159,593	256,270
% of mapped paired reads	96.93	95.81	82.41	95.97
Average contig length (bp)	933	678	515	773
Contig N_50_	2337	1099	617	1958
Non-redundant contigs	106,446	437,292	149,832	227,487
Total (predicted ORF)	17,034	64,670	62,271	42,922
Host (predicted ORF)	11,716	26,620	9008	21,297
Bacteria (predicted ORF)	97	10,274	27,035	N/A
Archaea (predicted ORF)	0	44	1045	N/A
Fungi (predicted ORF)	38	191	217	N/A
Ciliophora (predicted ORF)	0	0	1397	N/A
Nematoda (predicted ORF)	27	110	669	N/A
Others/unclassified ORF	5156	27,431	22,900	21,625

The BUSCO result agrees with ORF prediction as most of the genes represented in HG are of bacterial origin (3-folds higher ORF than host) (Table 1), and therefore host genes are possibly present in single copies. The number of host genes in FG, MG, and NG is comparatively higher than that of HG. As a result, the chance of having duplicated putative paralogous genes in our study is high as the Arthropoda genome represents an increased number of duplicated genes^63–65^, and we compared 1013 orthologous arthropod genes from 90 species in BUSCO (BUSCO:arthropoda_odb10). DE analysis revealed that FG and MG shared the highest number of expressed transcripts (29,557, i.e., 91.9%) while 491 and 2112 transcripts were unique to these segments, respectively (Fig. 1b).

MG and HG shared 24,338 (46.4%) transcript expression, and overexpression of 15,239 and 12,861 transcripts was unique, respectively. On the contrary, FG and HG shared the lowest number of transcripts, only 7209 (28.3%), while 1434 and 16,822 transcripts were unique to these gut segments. Together, all three gut segments shared the expression of 1434 (1.3%) transcripts. It indicates a higher abundance and diversity of microbial transcripts in HG than in other intestinal sections. ORFs were unequally distributed among three intestinal sections and taxa (host, Bacteria, Archaea, Fungi, Ciliophora, Nematoda) (Χ^2^(17) > 46,000, p < 0.0001, in all cases). Host-associated transcripts accounted for a fair portion of predicted ORFs in all studied body parts (Table 1). The asymmetric distribution of the host ORFs, with the maximum in MG (Observed vs Expected Frequencies, Χ^2^(3) = 11,812.10, p < 0.0001), followed by FG and HG, indicated differences in the activity of tissues along the intestinal tract. On the other hand, the expression of the microbial genes was the highest in the HG, followed by MG and FG. Thus, the contribution of host and microbial genes differed in distinct gut segments of the holobiont.

### Taxonomic origin of the functional microbiota in the millipede holobiont

**Figure 2:**
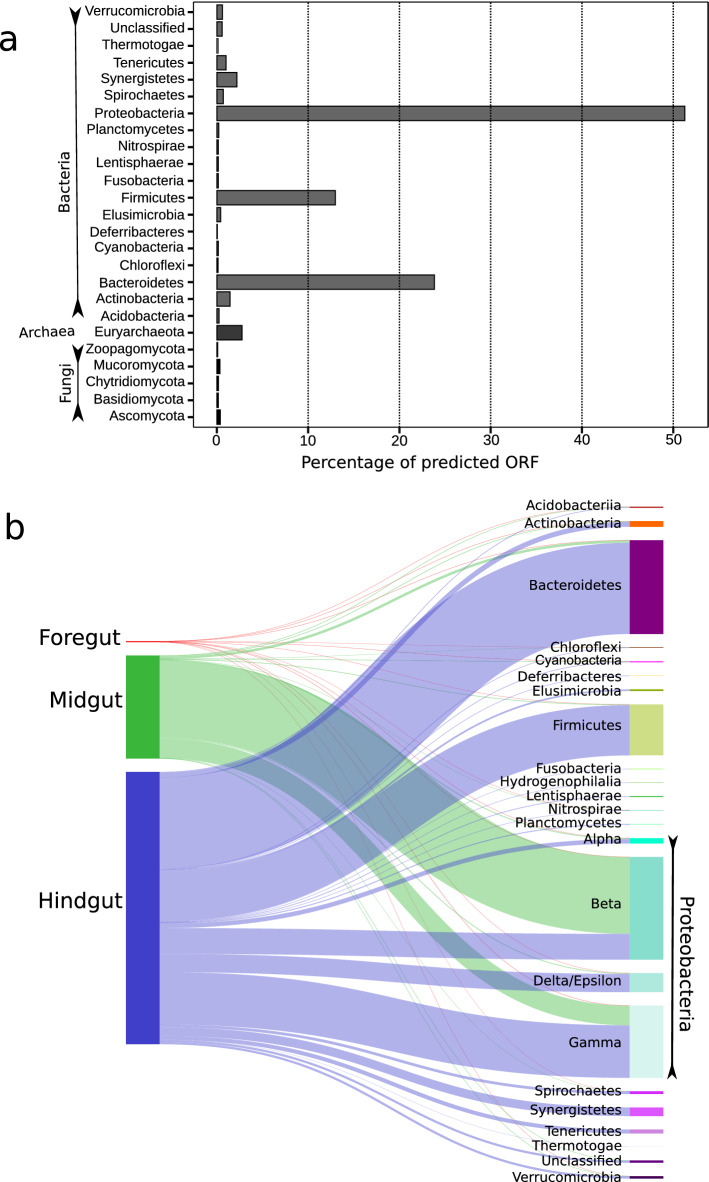
Taxonomic origin and distribution of functional microbiota in the millipede gut. (**a**) Bar plot showing the abundance of active microbiota in the millipede gut at the phylum level, assessed by the predicted ORF counts. (**b**) The abundance of functional bacterial phyla in the foregut, midgut, and hindgut of millipede species *T. aoutii*. The width of the connectors is proportional to the abundance of the taxa. Only the phylum Proteobacteria has been shown at the class level.

According to the taxonomic assignment of predicted ORFs, bacteria, archaea, fungi, and Ciliophora represented the active core host intestinal microbiota. Bacteria accounted for 92.7% of the total functional microbiome (Table 1) and were therefore referred to as the dominant host-associated microbiota in the millipede holobiont. Approximately 3.5%, 2.7%, and 1.1% of the total functional microbiome in the examined millipede specimens were represented by the ciliates, archaea, and fungi, respectively. Bacteria were mainly represented by Proteobacteria and Bacteroidetes followed by Firmicutes (Fig. 2a), accounting for 51%, 24%, and 13% of the microbial transcripts in the millipede holobiont, respectively. Many arthropods, including diplopods, harbor Firmicutes and Bacteroidetes in their gut, which contribute to the host's functional traits, including anaerobic metabolism, energy conservation, and methanotrophy^66^. Proteobacteria also dominates the microbial community in the gut segments of some insects^66,67^.

The dominance of Proteobacteria and Bacteroidetes among bacterial transcripts is typical in the leaf litter bacterial community (similar to the food source in the laboratory setup) from temperate and tropical deciduous forests (original food of *T. aoutii*)^68^. Such leaf litter is usually enriched in nitrogen and more easily degradable compounds such as cellulose and hemicellulose than soil, favoring bacterial decomposers. Many bacterial taxa of all three dominant phyla mentioned above decompose leaf litter in deciduous forest soils by cellulose-degrading activity^69,70^, suggesting that they may contribute to millipede gut digestive processes. In nature, leaf litter decomposing bacterial communities change successively as bacteria associated with the phyllosphere are replaced by other taxa over time, which is associated with an increase in bacterial community diversity^71^. Similarly, the shift in the composition of functionally active bacteria occurred along the intestine of *T. aoutii*, with a peak of Proteobacteria (mainly Beta- and Gammaproteobacteria) in MG (94% of the total bacterial transcripts), while diverse bacterial taxa were active in the HG, being dominated by Bacteroidetes, Firmicutes, Gamma-, Beta- and Delta/Epsilonproteobacteria followed by many others (Fig. 2b). 97% and 98.6% of the total active populations of Bacteroidetes and Firmicutes were present in the HG alone, respectively. This difference in the bacterial community may be due to the increase in pH between MG and HG. Such a shift in pH was reported in millipedes of the related species *E. pulchripes* and *A. gigas*^72^.

Functional bacterial population assessed by the ORFs increased along with the gut segments in a ratio of FG:MG:HG::1:106:279 (Observed vs Expected Frequencies Χ^2^(3) = 29,678.62, df = 2, p < 0.001), indicating that bacteria were the most active in the HG of *T. aoutii*, despite the absence of hindgut paunch in juliform millipedes^3^. The well-developed hindgut cuticle with spines and secretion of Malpighian tubules, containing buffer solution and nitrogenous wastes, facilitates microbial colonization^73^, while FG and MG prevent settlement of larger microbial populations by structure^3^ or secretion mechanisms^1^. All the archaea in the millipede gut belonged to the Euryarchaeota, represented by the Stenosarchaea, Diaforarchaea, and Methanomada (class I methanogens), accounting for 70%, 15%, and 9% of the total archaeal transcripts in the millipede holobiont, respectively. No active archaea were found in the FG, but most of the archaeal population was found in the HG, indicating the association of methanogens with the increased (plant material degrading) microbial activity. Methanogens in arthropod intestines are dependent on the delivery of methanogenic substrates by cellulolytic activity and subsequent anaerobic fermentation^74^ occurring in the HG.

Functionally active fungi were in the minority in the transcriptome, consisting mainly of Ascomycota (31% of total fungal transcripts), followed by Mucoromycota (29%), Chytridiomycota (14%), and Basidiomycota (13%). Different fungi were active in the distinct gut sections, e.g., Ascomycota and Mucoromycota transcripts dominated in the MG, while those of Chytridiomycota and Basidiomycota in the HG. In a previous study, based on cultivation efforts, some members of Ascomycetes, Basidiomycetes, and one species of Trichomycetes (recently included in Zoopagomycota) were documented in the midgut and hindgut of some millipedes (Julida, Glomerida, and Spirobolida^1^). Intestinal yeasts can actively contribute to the degradation of plant biomass due to their fermentative metabolism under O_2_ limiting conditions in the gut^1^. A more stable population of yeasts, mostly belonging to Ascomycetes might have survived gut passage and accumulated in the gut independently of feeding, as reported in previous studies^75^. Eukaryotic intestinal commensals or parasites Ciliophora and Nematoda were found mainly in the HG of *T. aoutii* which aligns with the previous study^3^.

### Gene function at the holobiont level

**Figure 3:**
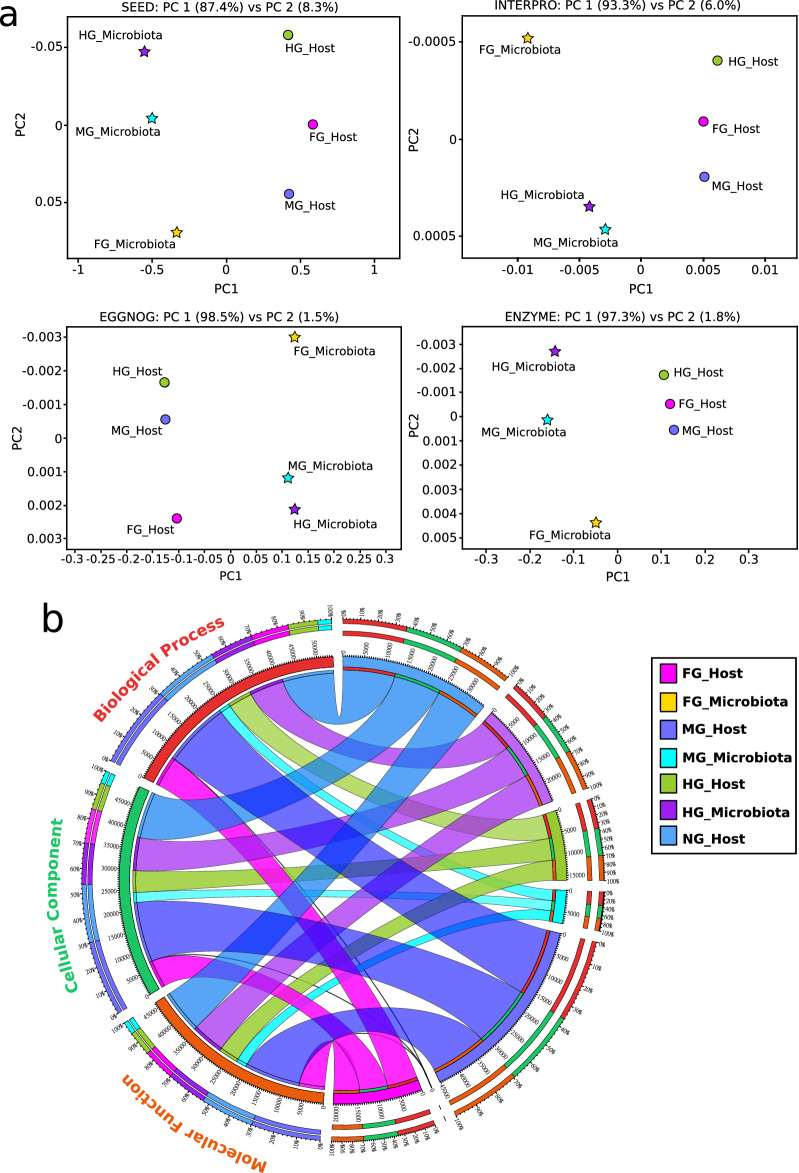
The functional genes in the millipede holobiont retained their taxonomic identity, and their contribution to the overall biological functions is different. (**a**) Principle coordinate analysis of functional genes from host and microbiota in the foregut (FG), midgut (MG), and hindgut (HG) using four functional databases, i.e. SEED, InterPro, eggNOG, and ENZYME. Genes belonging to host and microbiota are shown with circles and stars, respectively. (**b**) Circos plot representation of the distribution of genes belonging to the three most widely defined gene ontology (GO) categories (i.e. Biological Process, Cellular Component, and Molecular Function). GOs were plotted for the host as well as microbiota present in the foregut (FG), midgut (MG), hindgut (HG). Non-gut (NG) segment depicted GO expression in the millipede host only.

PCoA distinctively separated microbial and host transcripts from each other regardless of the databases used for functional assessment. This clustering explained a minimum of 87% of functional variability (Fig. 3a). Metatranscriptomic study of non-model holobionts represents bioinformatics challenges as the de novo assembly may introduce biases such as the formation of chimeric sequences resulting from the misassembly of RNA fragments from the host and the symbionts^76,77^. Using four independent databases at the functional level, we have demonstrated that our assembly was of good quality and that the active genes of host and symbiont origin were identified with high confidence.

We explored the gene ontologies (GO) associated with the identified protein sequences to obtain an overview of the active functional genetic diversity in different body parts and its distribution between host and associated microbiota. Abundance differences of GO terms among body parts highlighted the functional differences between compartments within the millipede holobiont (Fig. 3b). The highest functional diversity, expressed in GO terms, was shown by MG, where the host functions predominated (6455 host proteins), followed by the HG, where functions performed by microbiota prevailed (5349 proteins of microbiota), and NG, where only host functions occurred (Fig. 3b). Host genes active in the MG represented the highest number of attributions (45,197 in total), representing ~ 30% of the functionality in each class of the most general GO terms (i.e., “biological process” (GO:0008152), “cellular component” (GO:0005575), and “molecular function” (GO:0003674)). In a contemporary study on a wood-feeding termite, GO terms associated with the biological process, cellular process, and metabolic process were most abundant^78^. In the HG, genes of microbial origin accounted for ~ 16%, ~ 17%, and ~ 17% of the above-mentioned GO terms, respectively, while genes of host origin were only ~ 11% in each case. These results indicated that the biological functionality in the hindgut of millipedes is primarily governed by the microbial community, similar to a previous study on other arthropods^67^, and implies high functional diversity due to the microbial activity in the hindgut. Genes in the NG and FG contributed to ~ 23% and ~ 14% of the functionality at each functional level, respectively. The FG was predominated by the host genes, while genes of microbial origin represented < 0.05% of the total holobiont genes.

**Figure 4:**
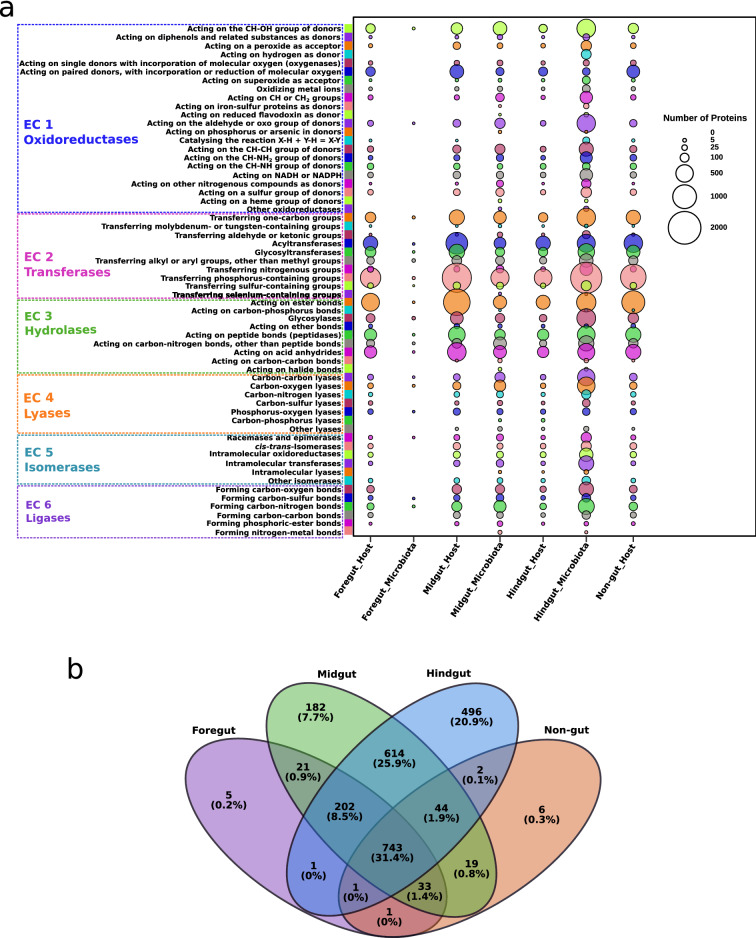
(**a**) Abundance and distribution of proteins, classified according to the enzyme classes in the millipede holobiont. The bubble size depicts the enzyme expression level. (**b**) Venn diagram of the distribution and abundance of enzyme classes at fourth level classification (i.e. EC x.x.x.x, “x” being any number according to the Enzyme Commission number) in different tissues in the millipede holobiont. Unique and shared enzymes are shown in number and percentage (up to one decimal).

Our holobiont transcribed 37,831 protein-coding genes corresponding to 2370 Enzyme Commission (EC) numbers. This unique dataset of enzyme class abundance in the holobiont allowed us to draw the first-ever picture of enzyme functioning in a millipede and its associated microbiota. The highest number of unique enzyme types in the HG of the millipede holobiont is linked with the higher microbial diversity. Our metatranscriptomic analysis revealed that although all the enzyme classes were ubiquitously produced in all gut sections (Fig. 4a), 496 (20.9%), 182 (7.7%), and 5 (0.2%) enzyme classes (i.e., EC numbers) were exclusively expressed in the HG, MG, and FG, respectively (Fig. 4b). A total of 743 enzyme coding transcripts (31.4%) were found in all gut segments as well as in the NG. A detailed description of the predicted ORFs belonging to the major enzyme classes (i.e., EC1-EC6) has been provided in Supplementary Table S3. The microbiota produced most of the enzymes in the hindgut, accounting for ~ 35% of all the identified enzymes. A similar study on wood-feeding termites showed that most of the lignocellulose degrading enzymes were expressed by the microbiome in the hindgut^10^ possibly due to the rich and complex microbial community in the hindgut^79^. The host produced about 21% of the enzymes in the midgut, while the non-gut tissues produced approximately 15% of the enzymes. Among all the enzymes, transferases were the most abundant (14,068 proteins, 37.19%), followed by hydrolases (10,349 proteins, 27.35%) and oxidoreductases (6956 proteins, 18.39%). The abundance of more specific GO terms and the genes encoding the enzymes involved in selected functional traits are listed in Supplementary Tables S4–S11. Comparative analyses of clusters of orthologous groups (COGs) in the millipede host and associated microbiota revealed a differential distribution of functional genes among body segments in the host and microbiota (Supplementary Fig. S1) and corroborated with the results above. Although function (COG) such as “amino acid metabolism and transport” was performed by both the host and the microbiota, the involvement of the microbiota in this process was significantly higher than the host.

### The role of microbiota in the digestive processes of the host

Genes involved in millipede digestive processes indicated by the GO terms and COG categories were mainly expressed in the MG by the host (Supplementary Fig. S1 and Table S4), which agrees with the primary role of the millipede midgut in digestion. Soluble nutrients are passed through the peritrophic membrane in the millipede midgut and assimilated via the microvilli^3^. Nutrients are also released after digestion by enzymes derived from the secretions of the salivary glands and midgut epithelium. Enzymes pass from the midgut cells through the peritrophic membrane and may be supplemented by microbial enzymes in the midgut lumen^3^. Measurements of enzyme activity in homogenates of different intestinal sections of spirostreptid and spirobolid millipedes revealed high activity of polysaccharidases in the midgut in a previous study^2^.

Host digestive activities are also expected in salivary glands sampled together with the FG, whereas the HG is a site of water and mineral reabsorption, and only some secretion activity was previously documented^2^. Our previous study on enzyme quantification in the midgut and hindgut homogenates of the spirostreptid millipede *A. gigas*^2^ suggested a complete cleavage of the substrate by intestinal homogenate without specifying the origin of the enzymes. Metatranscriptome analysis showed that the diversity of expressed enzyme-coding genes in a body compartment may not directly reflect the actual enzymatic activity (Supplementary Table S4). Few, mainly autochthonous enzymes in the midgut may have higher overall activity than a variety of diverse microbial enzymes in the hindgut. Millipede genes encoding for digestive enzymes were also active in non-intestinal tissues (NG), indicating their alternative, non-digestive role. Digestion of lipids, proteins, α-polysaccharides, sucrose, and chitin took place in the midgut, catalyzed by autochthonous millipede enzymes, secreted by midgut epithelium or salivary glands, with partial involvement of bacterial enzymes (Supplementary Table S5).

The active host genes for laminarinase and trehalose were not detected in this study. Both were detected only in the HG and identified as microbiota-associated, even though a previous study demonstrated the degradation of trehalose and laminarin in the midgut homogenate of another spirostreptid millipede species *A. gigas* using enzymatic assays^2^. We did not detect active genes encoding for enzymes with the potential to degrade lignin. Superoxide dismutase, which has been detected in the host, ciliates, and bacteria in the holobiont (mainly in the hindgut), is a vital antioxidant that protects all living cells exposed to oxygen. We found host-associated putative lignin modifying enzyme hemocyanin^80^ in our metatranscriptomic data, a copper metalloprotein which may act as an oxygen carrier^81^. This enzyme belonging to the tyrosinase group was expressed in the host, ciliates, and bacteria and may facilitate lignin degradation in arthropods (Supplementary Table S5).

### Aerobic and anaerobic energy metabolism in the holobiont

Anoxic conditions in the midgut and hindgut of spirostreptid millipedes have already been reported^72^. Anoxia in arthropod guts results from the activity of the facultative anaerobic microbiota depleting oxygen from the gut content^82^. It implies the existence of a thin microxic layer at the internal surface of the intestinal wall. It agrees with the simultaneous presence of aerobic and anaerobic metabolic types (GO terms “aerobic respiration” and “anaerobic respiration”) in the intestinal bacterial community (Supplementary Table S6). The distribution of essential enzymes involved in aerobic respiration is shown in Supplementary Table S7.

The anoxic intestinal content provides conditions for fermentative processes indicated by the GO term “fermentation” and the filial terms and by the chimeric metabolic pathways of microbial communities. These results show different types of fermentation carried out mainly by the microbiota in both MG and HG (Supplementary Table S6). A mixture of short-chain fatty acids (SCFAs) and gases (i.e., hydrogen, carbon dioxide, and methane) are likely products of fermentation pathways indicated by expressed genes in the gut of *T. aoutii*. Eukaryotic commensals (at least the ciliates) in the hindgut can contribute to by-products of their anaerobic metabolism (GO term “anaerobic respiration” in Supplementary Table S6). Methanogenesis performed by archaea is driven by the production of fermentation products and facilitated by oxygen limitation in the hindgut (Supplementary Table S6). The presence of SCFAs and hydrogen in the midgut and hindgut and methane production in the hindgut have been previously confirmed in two different millipede species^72^.

### Energy utilization and storage in the holobiont

The presence of COG “lipid transport and metabolism” and some GO functional terms indicate the activity of genes supporting nutrient absorption in both the midgut and hindgut (Supplementary Fig. S1 and Table S4). In some millipedes, indentations over the cuticular surface of the hindgut serve as possible channels for nutrient exchange between the hindgut lumen and the hemolymph^73^. Such channels could allow millipedes to utilize microbial fermentation products that accumulate in the hindgut. Uptake of bacterial fermentation products from the hindgut has been confirmed in other arthropods^83^. Absorbed nutrients are stored in the intestinal epithelium and non-intestinal tissues of the millipede in the form of storage carbohydrates (glycogen) and storage lipids (see GO terms associated with “energy reserve metabolic process” and associated functions in Supplementary Table S8). Analysis of GO terms indicated that millipede carbohydrate homeostasis may be regulated similarly to insects and that polysaccharides accumulated in tissues are recycled by phosphorolysis driven by phosphorolytic enzymes (Supplementary Tables S7 and S8).

Millipedes accumulate structural cuticular polysaccharides. Because chitin is the primary component of the arthropod cuticle, genes involved in its metabolism are represented by millipede transcripts that are ubiquitously present in all gut and non-gut tissues (Supplementary Table S8). Chitinolytic enzymes in the holobiont can be digestive or non-digestive and play a role in host cuticle reconstruction (Supplementary Tables S7 and S8). Some chitinases with broader substrate specificity can have additional antibacterial and antifungal importance^84^. On the other hand, bacterial chitinases are likely involved in later stages of leaf litter decomposition when bacteria feed on fungi^71^; however, this probably does not occur in the millipede intestine as only a few were detected. In our study, activities related to glycogen and lipid storage were detected in bacteria (Supplementary Table S8, GO:0052576, and GO:0019915). Storage compounds from midgut bacteria can represent a potential nutrient source (storage compounds and nitrogen) for millipedes, as discussed elsewhere^85^. Leaf litter is a relatively low-nitrogen diet for millipedes, and microorganisms may play a crucial role in arthropod’s nutritional nitrogen requirements. Nitrogenase genes were active in hindgut bacteria, but functional GO terms “nitrogen fixation” and “nitrogenase activity” were not found in the data (Supplementary Table S9). Computational prediction of nitrogen fixation by microbes is very sensitive to the correct annotation and databases and the associations of these databases with the gene ontology database^86^. Therefore, our analysis of general transcriptomics might have missed those nitrogen fixation GO terms that could be detected by a more targeted analysis and are currently beyond the scope of this study.

### Intestinal microbiota as a source of essential compounds for the host

**Figure 5:**
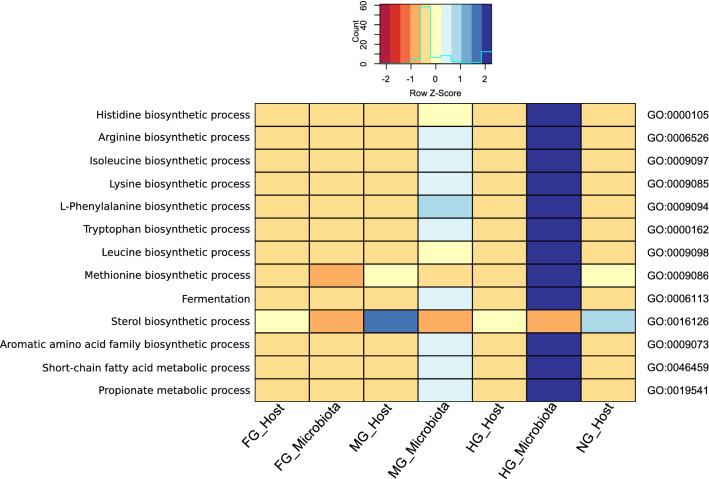
Heat map of the selected GO terms, especially amino acid biosynthetic processes in the assembled millipede holobiont. The colors of the scale bar denote the level of abundance on the Z-score, with dark blue and dark red indicating the highest and lowest level of abundance, respectively. The samples from foregut (FG), midgut (MG), hindgut (HG), and non-gut (NG) were compared.

In addition to dietary supply, the intestinal microorganisms synthesized essential compounds (biotin, thiamin, and folic acid) and some amino acids for the host (Supplementary Table S10). This study revealed that millipedes can synthesize ascorbate with autochthonous l-gulonolactone oxidase (Supplementary Table S9, EC 1.1.3.8) and vitamin K (Supplementary Table S10, GO:0042371). All millipede tissues and ciliates examined in our study contain the active gene for coding very-long-chain 3-oxoacyl-CoA synthase (Supplementary Table S9, EC 2.3.1.199), which enables the host to perform some steps in the biosynthesis of unsaturated fatty acids (Supplementary Table S10, GO:0006636). However, aromatic amino acids (phenylalanine, tryptophan, and tyrosine) can only be synthesized by plants and microorganisms^87,88^, whereas animals rely on their diet to obtain these amino acids. We have shown that in our millipede holobiont, the “aromatic amino acid family biosynthetic process” was carried out exclusively by the microbiota in the midgut and hindgut (Fig. 5). In fact, GO terms associated with many of the amino acids were predominantly enriched in the hindgut microbiota. Designated enzymes required for phenylalanine, tryptophan, and tyrosine biosynthesis were exclusively present in the microbiota (Supplementary Figs. S2 and S3). Histidine, the precursor of histamine that acts as a neurotransmitter in invertebrates, was also produced only by microbiota and contained the complete set of enzymes for its biosynthesis (Supplementary Fig. S4), in contrast to the millipede host (Supplementary Fig. S5). A list of the detected expressed enzymes with diverse biological functions has been provided in Supplementary Tables S9 and S10.

### Defense against intestinal parasites and pathogens

The distribution of GO terms indicating “responses” to other organisms illustrates microbial interactions within the holobiont (Supplementary Table S11). Millipede defense responses are not concentrated in the intestine despite having hindgut populations of ciliates and nematodes with unclear symbiotic statuses (commensals, parasites, or pathogens). Microscopic fungi can act as pathogenic factors for millipedes, and genes involved in defense against fungi were active in all host body parts. In a recent study, eight species of antibiotic-producing Actinobacteria belonging to the genus *Streptomyces* were detected in the digestive tract of millipede *Nedyopus dawydoffiae* (Diplopoda) (Attems, 1953)^89^. We have found that lysozyme genes were produced by the host tissues as a part of probable antibacterial defense. Microbivory is also a natural defense mechanism in millipede which is performed by ciliates and nematodes primarily in the hindgut. The defense glands of *T. aoutii* produce a mixture of hydroquinone derivatives^12,90^. The biochemical process related to their biosynthesis is probably not specifically included in the gene ontology database and therefore was not precisely annotated in this study. The terms “quinone biosynthetic process” and “quinone metabolic process” (GO:1901663, GO:1901661, Supplementary Table S11), are common in all parts of the millipede body, and may partly reflect it. Rodriguez et al. used several transcriptomics data in their phylogenomics study to demonstrate that hydroquinone and benzoquinone are two ancient chemical forms that millipedes use for chemical defense^12^.

This study provided the first-ever molecular picture of gene expression at the holobiont level in the Myriapoda. Numerous aspects led us to affirm that gene function in millipedes occurs at the holobiont level with synergistic contributions from the host and microbiota, and therefore non-model species like the millipede host could serve as a promising model for holobiont studies. The diversity and abundance of many essential genes important for maintaining the biological functions of the millipede holobiont were produced differently by the host and microbiota in distinct gut segments. Microbiota predominantly contribute to functions such as metabolism of crucial amino acids, SCFA, and fermentation, while the host genes govern biological processes like sterol biosynthesis. As the millipedes in this experiment were reared in the laboratory, we could not draw a conclusion whether environmental variation causes changes in gene expression at the holobiont level, but the presence of a diverse set of genes indicates that this potentially occurs. A different picture could emerge under different environmental conditions, such as habitat, food sources, and chemical soil pollution, which remain to be explored. Due to the unavailability of a reference genome, the assembly was performed de novo*.* This should be considered a valuable resource as various quality checkpoints were set throughout the analysis and checked with genome alignment when available or through in vitro validation (e.g., PCR) when considering a restricted group of functions.

## Supplementary Information

Supplementary Information.

Supplementary Tables.

## Data Availability

The sequence files generated in this study were deposited to NCBI’s Sequence Read Archive (BioProject PRJNA749320) with the accession numbers SRR15239520 to SRR15239535.
