# Supplementary material for: De novo metatranscriptomic exploration of gene function in the millipede holobiont
Source: Sci Rep. 2022 Sep 28;12:16173. doi: 10.1038/s41598-022-19565-y (PMC9519908; doi:10.1038/s41598-022-19565-y)
Supplement: Supplementary file 1 — Supplementary Information. [file 41598_2022_19565_MOESM1_ESM.pdf]

De novo metatranscriptomic exploration of gene functioning in millipede holobiont

Puspendu Sardar[1,2] *, Vladimír Šustr[1], Alica Chroňáková[1], František Lorenc[1,3], Lucie Faktorová[1]

[1]Institute of Soil Biology, Biology Centre AS CR, Na Sádkách 7, CZ 370 05, České, Budějovice, Czech Republic

[2]Cambridge Institute of Therapeutic Immunology & Infectious Disease (CITIID), Department of Medicine, Cambridge Biomedical Campus, University of Cambridge, Cambridge CB2 0AW, United Kingdom

[3]Department of Food Biotechnologies and Agricultural Products' Quality, Faculty of Agriculture and Technology, University of South Bohemia in České Budějovice, Studentská 1668, CZ 370 05, České Budějovice, Czech Republic

*Corresponding author: Puspendu Sardar; email: puspendu.sardar@gmail.com

Supplementary material

Table S1: Total amount of extracted RNA and RNA integrity number (RIN) for each sample in the study. N/A – not available.

Sample	Individual	Body part	Total RNA (μg)	RIN
TA3_FG	No. 3	foregut	0.682	N/A
TA3_MG	No. 3	midgut	0.748	4.8
TA3_HG	No. 3	hindgut	0.582	4.2
TA3_NG	No. 3	non-gut	1.562	N/A
TA4_FG	No. 4	foregut	0.814	N/A
TA4_MG	No. 4	midgut	0.638	N/A
TA4_HG	No. 4	hindgut	0.924	5.1
TA4_NG	No. 4	non-gut	1.562	5.9
TA7_FG	No. 7	foregut	0.616	N/A
TA7_MG	No. 7	midgut	0.264	6.9
TA7_HG	No. 7	hindgut	15.18	N/A
TA7_NG	No. 7	non-gut	1.034	7.7
TA8_FG	No. 8	foregut	0.902	8
TA8_MG	No. 8	midgut	0.638	6.2
TA8_HG	No. 8	hindgut	5.104	4.8
TA8_NG	No. 8	non-gut	0.462	6.7

Table S2: Reads statistics of the unassembled metatranscriptomics datasets of the millipede species *T. aoutii*

	Foregut (Number of PE Reads)		Midgut (Number of PE Reads)		Hindgut (Number of PE Reads)		Non-gut (Number of PE Reads)	
Biological Replicates	rRNA	Putative mRNA	rRNA	Putative mRNA	rRNA	Putative mRNA	rRNA	Putative mRNA
TA3	18,787,239	10,659,844	12,718,909	15,722,301	48,939,820	4,157,522	17,218,518	10,833,809
TA4	13,135,451	13,449,149	11,066,858	17,448,623	47,943,438	5,529,056	11,078,612	16,486,981
TA7	15,918,158	12,355,657	10,353,718	13,486,295	40,058,759	5,184,926	11,397,099	17,282,064
TA8	20,687,045	12,961,369	10,533,075	16,705,289	52,730,768	3,762,448	22,150,334	10,757,605
Average	17,131,973.2	12,356,504.8	11,168,140	15,836,127	47,418,196.2	4,658,488	15,461,143.2	13,841,115
Total	68,527,893	49,426,019	44,672,560	63,362,508	189,672,785	18,633,952	61,844,563	55,360,459

Table S3: Number of predicted ORFs belonging to different enzyme classes

	FG Host	FG Microbiota	MG Host	MG Microbiota	HG Host	HG Microbiota	NG Host
Oxidoreductases (EC 1)	480	3	958	1156	396	3177	786
Transferases (EC 2)	1499	14	3368	1342	1179	4132	2534
Hydrolases (EC 3)	1169	11	2710	1062	803	2636	1958
Lyases (EC 4)	109	4	224	454	100	1272	203
Isomerases (EC 5)	81	1	215	236	97	1030	141
Ligases (EC 6)	164	3	358	473	115	948	230
Total	3502	36	7833	4723	2690	13195	5852
Total percentage (%)	9.2	0.1	20.7	12.5	7.1	34.9	15.5

Figure S1: Heat map of the clusters of orthologous groups (COGs) in millipede holobiont. The colors of the scale bar denote the level of expression on the Z-score, with dark blue and dark red indicating the highest and lowest level of expression, respectively. The samples from foregut (FG), midgut (MG), hindgut (HG), and non-gut were compared.

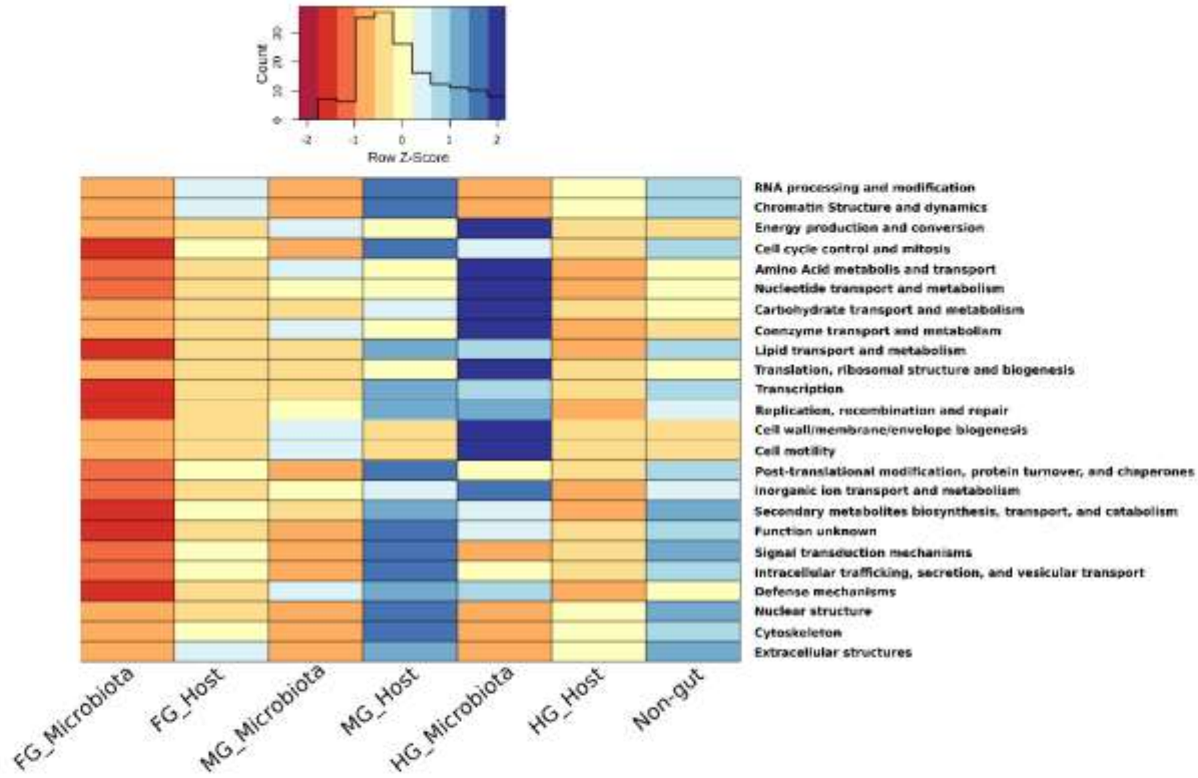

Figure S2: **Microbial biosynthetic pathway of phenylalanine, tyrosine, and tryptophan**. The phenylalanine, tyrosine, and tryptophan biosynthetic pathway and known E.C. numbers of the enzymes involved from KEGG[1-4]. The biosynthetic pathway has been marked with pink lines. The enzymes detected at a high level in the microbial transcriptomes in all the gut segments are indicated in green boxes. In addition to the phenylalanine, tyrosine, and tryptophan biosynthetic pathway, the shikimate pathway is also active in microbiota in millipede holobiont to produce aromatic amino acids.

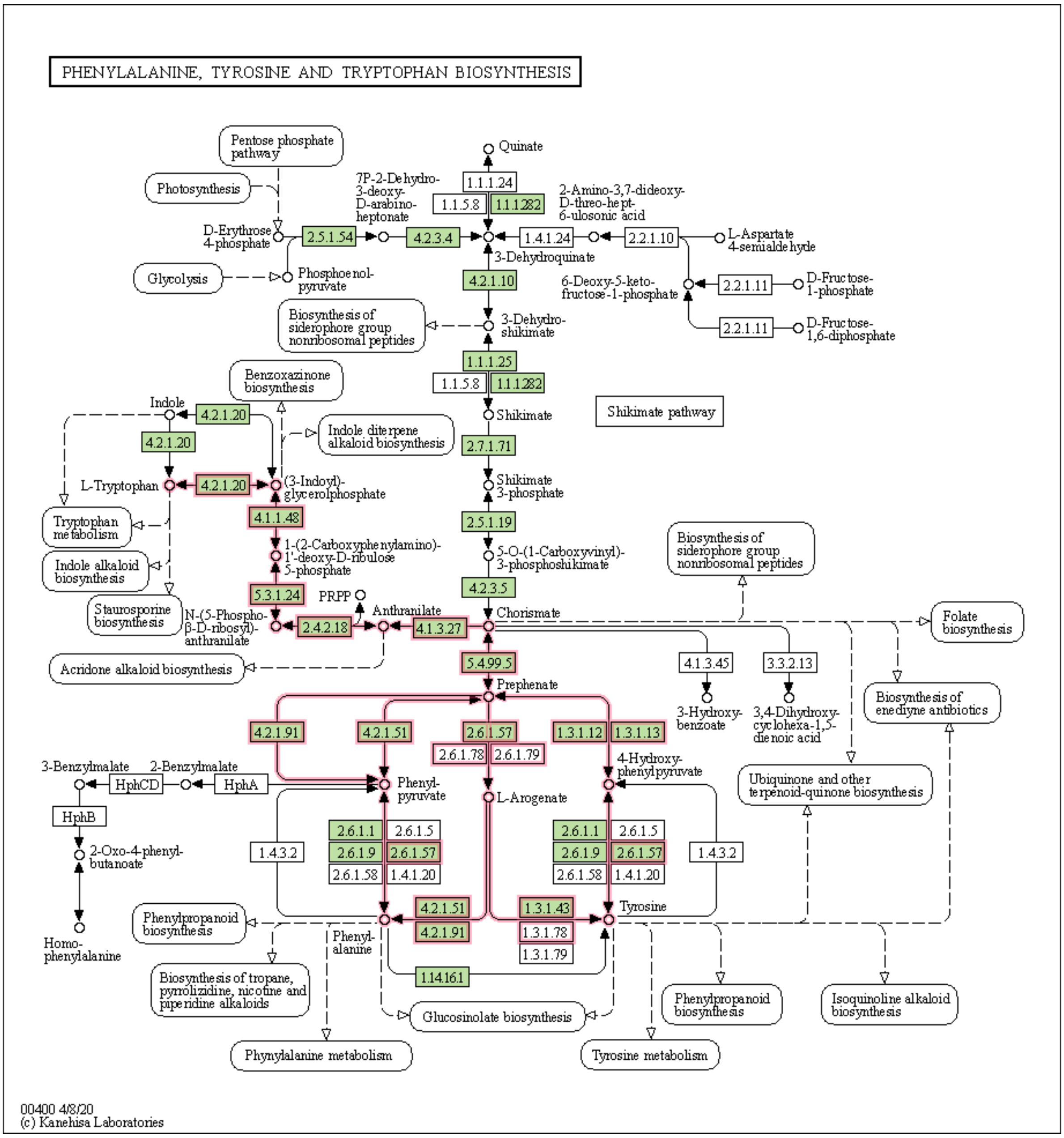

Figure S3: Host biosynthetic pathway of phenylalanine, tyrosine, and tryptophan. The phenylalanine, tyrosine, and tryptophan biosynthetic pathway and known E.C. numbers of the enzymes involved from KEGG[1-4]. The biosynthetic pathway has been marked with pink lines. The enzymes detected at a high level in the host transcriptomes from all the body parts are indicated in magenta-colored boxes. A very few enzymes that might have other physiological functions are active in the pathway. Most of the enzymes in the pathway are absent, and hence the overall pathway in the host seems inactive.

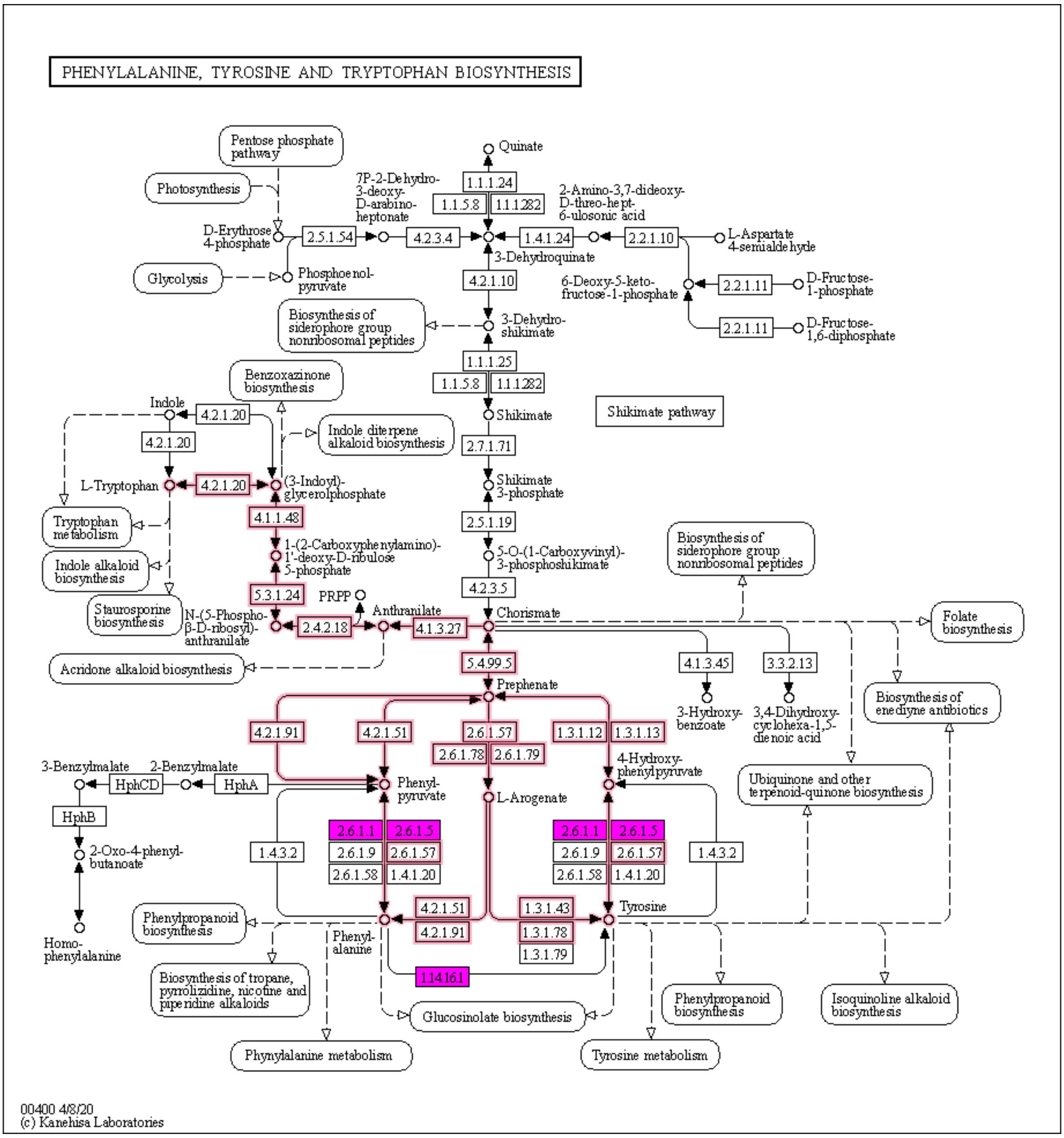

Figure S4: Microbial biosynthetic pathway of histidine. The histidine metabolic pathway and known E.C. numbers of the enzymes involved, from KEGG[1-4]. Histidine biosynthetic pathway is highlighted with pink lines. The enzymes detected at a high level in the microbial transcriptomes are indicated in green boxes. Both the biosynthetic and catabolic enzymes of the pathway are present in the microbiota of the millipede holobiont.

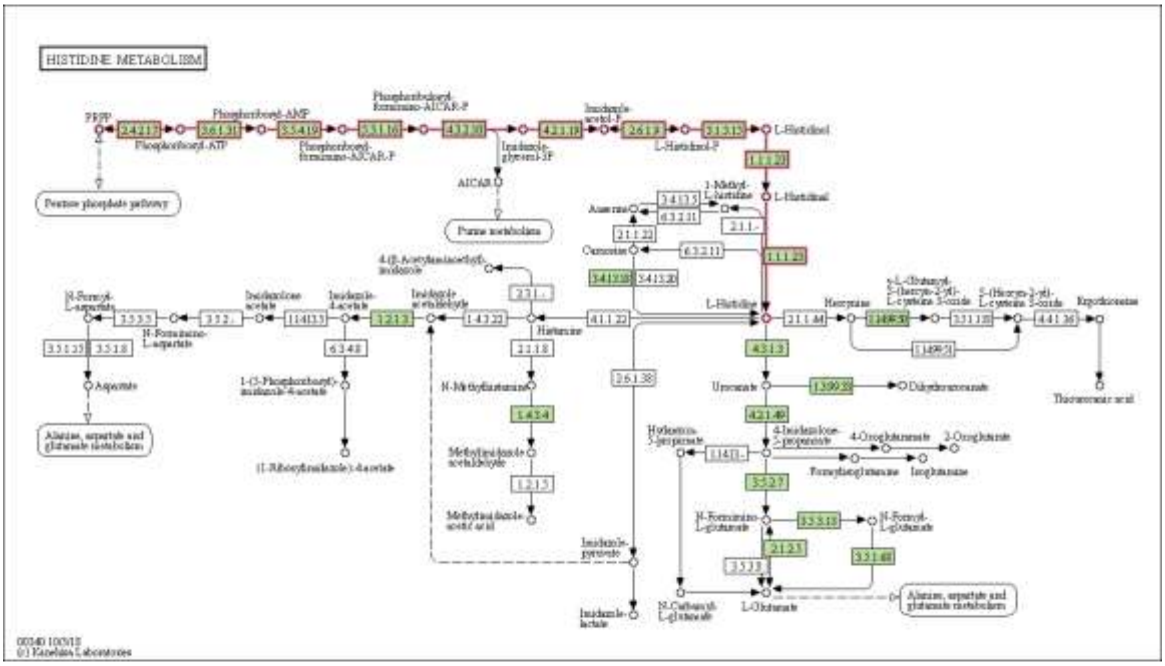

Figure S5: Histidine biosynthetic pathway in the host. The histidine metabolic pathway and known E.C. numbers of the enzymes involved, from KEGG[1-4]. Histidine biosynthetic pathway is highlighted with pink lines. The enzymes detected at a high level in the host transcriptomes are indicated in magenta-colored boxes. Only the catabolic enzymes of the pathway are present in the millipede host.

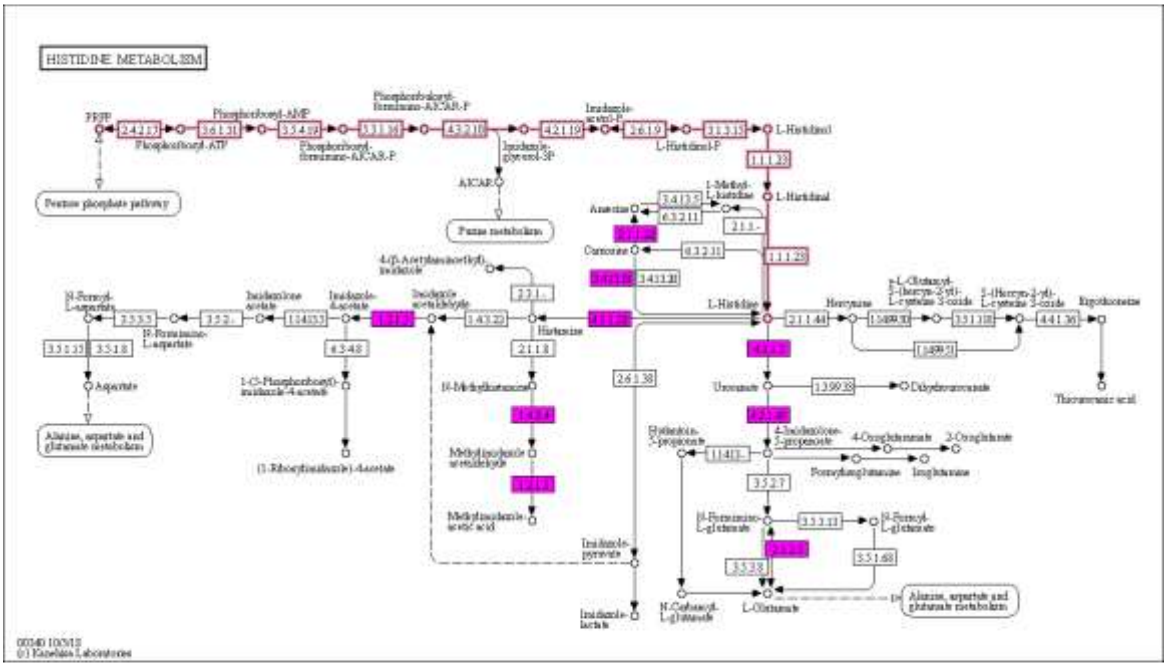

Reference:

1 Kanehisa, M., Furumichi, M., Sato, Y., Ishiguro-Watanabe, M. & Tanabe, M. KEGG: integrating viruses and cellular organisms. *Nucleic Acids Res* **49**, D545-D551 (2021).

2 Kanehisa, M. & Goto, S. KEGG: Kyoto Encyclopedia of Genes and Genomes. *Nucleic Acids Res* **28**, 27-30 (2000).

3 Kanehisa, M. & Sato, Y. KEGG Mapper for inferring cellular functions from protein sequences. *Protein Sci* **29**, 28-35 (2020).

4 Kanehisa, M. Toward understanding the origin and evolution of cellular organisms. *Protein Sci* **28**, 1947-1951 (2019).
